# Workplace health promotion interventions for nurses in Germany: a systematic review based on the RE-AIM framework

**DOI:** 10.1186/s12912-022-00842-0

**Published:** 2022-03-21

**Authors:** Andrea Schaller, Madeleine Gernert, Teresa Klas, Martin Lange

**Affiliations:** 1 Working Group Physical Activity-Related Prevention Research, Institute of Movement Therapy and Movement-Oriented Prevention and Rehabilitation, German Sport University Cologne, Cologne, Germany; 2 Department of Fitness and Health, IST University of Applied Sciences, Erkrather Straße 220 a-c, 40233 Düsseldorf, Germany

**Keywords:** Workplace health promotion, Healthcare, Longterm care facilities, Acute medical hospitals, Home-based longterm care, Nursing profession

## Abstract

**Background:**

The German health care system is faced with a serious shortage of nurses. This is associated, amongst other things, with difficult working conditions and work-related health burdens. Workplace health promotion (WHP) is considered a promising approach to promote the health of nurses. The present review aims to give an overview on existing interventions in different nursing settings (acute care hospitals, long-term care (LTC) facilities and home-based long-term care) in Germany.

**Methods:**

A systematic literature search was conducted in PubMed and PubPsych. Studies were included if published after 2010 and provided data of intervention studies on workplace health promotion among nurses in Germany (RCTs, non-RCTs, non-controlled intervention studies and pilot studies). The setting in which the study was conducted (acute medical care hospital, inpatient LTC facilities, home-based LTC, cross-setting) as well as the health issue assessed (physical health, mental health and/or violence experience) were extracted. The intervention was reported against the background of the quality criteria for prevention measures of the statutory health insurers in Germany. The results of the studies were presented according to the RE-AIM framework.

**Results:**

Eleven studies on WHP for nurses were included, whereof seven studies were conducted in acute medical care hospitals and four in LTC facilities. No study reported results on WHP for nurses working in the setting of home-based LTC. Most studies aimed at improving mental health. The intervention contents and forms of implementation were heterogeneous. According to the RE-AIM criteria, the reporting of most studies showed several limitations, especially a lack of reporting on *Implementation* and *Adoption*. Most studies showed no statistically significant effect on the respective outcomes *(Effectiveness)*. Four studies reported results on *Maintenance* indicating a sustained effectiveness.

**Conclusion:**

Despite the high relevance for health promotion for nurses, our review showed a striking lack of intervention studies in this field. From this we derive a high need of tailored interventions, taking into account the setting-specific development, implementation of WHP interventions for nurses. With regard to the evaluation, the RE-AIM criteria should be taken more into account in order to meet the requirements of evaluating complex interventions and thus contribute to evidence development of WHP in nursing. In terms of content, the topic of violence prevention and dealing with experiences of violence should also be taken into account. Regarding the settings, the working conditions and health burdens in LTC facilities, home-based LTC and acute medical hospitals must be considered.

**Trial registration:**

PROSPERO registration number: CRD42021231891

## Introduction

The growing shortage of professional nurses is a significant socio-political and healthcare issue [1, 2]. Nevertheless, the German health care system is faced with a serious shortage of skilled workers [1, 2] and especially the nursing profession appears to be unattractive [3]. Amongst other things, this is attributed to difficult working conditions (high work loads, shift work, time pressure, etc.) and occupational health burdens [4–9].

The current state of research indicates a greater health burden for nurses compared to other occupational groups (e.g. computer science, information and communication technology occupations, manufactoring industry) [10], for example a high prevalence of musculoskeletal complaints, reported to be in the range of 64–80% [11–13]. In addition, nursing staff shows a high prevalence in chronic stress [14] what also might be associated to further mental health problems, such as emotional exhaustion and burnout [15–17]. As several studies showed, that nurses are regularly exposed to verbal and physical violence, also including sexual harassment [18–21], the topic of dealing with or preventing experiences of violence is also of growing importance.

However, for a differentiated consideration of the work-related health burdens of nurses, the respective setting should be taken into account. In the German health care system, a basic distinction is made between outpatient and inpatient care. Both, medical care and long-term care (LTC) can basically be provided in an outpatient (e.g. medical practice) or inpatient setting (e.g. acute medical care hospitals) [22]. The most obvious difference between the settings is that inpatient care includes accommodation and meals. Inpatient LTC in Germany is provided, for example, in LTC facilities for the elderly or the disabled, whereas outpatient LTC can be provided in the patient's home environment or in specialised assisted living facilities [23]. With regard to nursing as a professional field, it is noticeable that, in 2019, the majority of nurses by far work in inpatient LTC (796,000), followed by acute medical care hospitals (450,000) and outpatient LTC (420,000) [24, 25]. Assuming that the professional activities of a nurse differ in part considerably depending on the care setting, the current state of research shows that this also could be related to different work-related health burdens [4, 26, 27]. Although the data on setting-based comparisons of health burdens is limited, available data indicate that nurses in acute medical care hospitals might be more likely to be affected by mental health problems [14] and nurses working in inpatient LTC facilities appear to be more frequently affected by experiences of violence, compared to nurses working in acute medical care hospitals and home-based LTC [20, 21].

Despite the lack of setting-specific data, the data on health burdens in nursing is fundamentally strong. Overall, workplace health promotion (WHP) is considered a promising setting promoting mental and physical health [28–31], which is also reflected in the Preventive Health Care Act in Germany [32]. In consequence, WHP has also become increasingly important in the nursing sector in recent years [33–35]. In general, WHP interventions are considered a promising approach in the promotion of health and well-being at work [28], as well as healthy behaviour (e. g. physical activity, dieatary habits) [36, 37]. On this basis, the *Care Staff Strengthening Act *[38] requires German statutory health insurers to spend one euro per insured person for WHP interventions in nursing care. Nevertheless, WHP still seems to be little established in nursing: Both at the employee level (47.5%) [10] and at the management level (43%) [2], less than half of the respondents stated that a WHP offer was available in their institution. There is also little specific knowledge about the challenges of implementing WHP for nurses in specific care settings, especially for outpatient LTC [35]. Therefore, the research questions of the present review were: 1) Which workplace-related health promotion interventions in acute medical care hospitals, inpatient LTC and outpatient LTC are available?, 2) How can the available interventions be appraised according to the RE-AIM framework?

## Methods

This systematic review was conducted following the international guidelines established by PRISMA (Preferred reporting items for systematic reviews and meta-analysis protocols) [39] and was registered in the International prospective register of systematic reviews (PROSPERO, registration number: CRD42021231891).

### Search strategy

The electronic databases PubMed and PubPsych were searched on January 11th, 2021 for potential articles. Search terms used for relevant studies were (nurs* OR "professional care" OR "professional caregiver") AND ("workplace health promotion" OR "work health promotion" OR WHP OR WHPP OR prevention OR “preventive health program" OR "preventive health care" OR "intervention program") AND (health* OR violence* OR "work ability" OR disease OR morbidity OR "risk factor" OR burden OR stress) AND (german*). Original studies in German or English language, published between January 01^st^, 2010 and January 11^th^, 2021 were taken into account. Results were completed by a manual search upon included studies’ references.

### Inclusion and exclusion criteria

In our review we defined workplace health promotion as behavioural measures offered at the workplace, addressing individual coping skills in the field of physical-activity-promoting work and physically active employees, stress-management and -strengthening resources, healthy diet in everyday work, and addiction prevention [40]. Studies which met the following inclusion criteria were examined: (1) target group or subgroup analysis: professional nurses in Germany, (2) setting: acute medical care hospital, inpatient LTC facilities and/or home-based LTC, (3) intervention study (RCTs, non-RCTs, non-controlled intervention studies and pilot studies), (4) primary outcome: physical health, mental health and/or violence experience. Articles that showed at least one of the following exclusion criteria were not considered for further analysis: (1) no original study (e.g. review or editorial), (2) interventions that were prilimilary addressing health and safety protection at the workplace (according to social code (SGB VII)), as another recognized pillar of a holistic workplace health management in Germany, (3) studies outside of Germany. Comparators were not defined in advance.

### Study selection, data extraction and synthesis

The study selection process after the elimination of duplicates was conducted with the software tool for systematic reviews “Rayyan” [41]. Two authors (MG, TK) independently performed the title and abstract screening as well as the subsequent full-text screening including the record of reasoned exclusion. Any discrepancies were resolved by discussion and consensus with a third researcher (AS). The selection process was illustrated in a PRISMA Flow Chart [39]. Data extraction of the included articles was separately performed by two authors (MG, TK) and crosschecked in each case.

In order to answer research question 1 on the setting specific availability of WHP for nurses, extracted data of the studies included were author and publication year, the setting in which the study was conducted (acute medical care hospital, inpatient LTC facilities, home-based LTC, cross-setting) and the health issue addressed in the study (physical health, mental health and/or violence experience), In addition, the interventions were presented against the background of the quality criteria for prevention measures of the statutory health insurers [40]. The quality criteria include planning and concept quality (target group; content; participants material; theoretical framework/evidence of the intervention), process quality (group size, contraindications, number, duration and frequency of units, location) and structural quality (provider qualification).

To answer question 2 on the appraisal of the respective studies, the study design and the comparators (ususal care, non-intervention, comparison intervention, no control group) were extracted and the results of the studies were presented according to the RE-AIM framework [42, 43]. Table 1 shows the chosen indicators for each RE-AIM dimension that were extracted in the present review. Missing information in the original studies on one dimension was described as "not reported".

**Table 1:** Operationalization of the RE-AIM dimensions in the present review

Dimension	Operationalization
Reach (individual level)	sample size, participants’ age and sex at baseline
Effectiveness (individual level)	the impact of the intervention on the primary outcome of the study
Adoption (organisational level)	number of participating organisations (settings)
Implementation (organisational level)	availability of information on the extent to which the program is delivered as intended
Maintenance (individual and organisational level)	longterm effects of the program on primary outcomes after the intervention

### Quality assessment

The Delphi List [44] was applied in order to evaluate the selected articles and to identify the risk of bias of the included studies. The Delphi List consists of nine items, which are answered with "yes", "no" or “don’t know”. Two authors (MG, TK) independently applied the checklist. In case of disagreements in the ratings of the nine items, they were resolved after reconsideration and, if necessary, discussed with a third author (AS). Finally, the percentage of checklist items answered with “yes” was calculated for each study. If the study scored ≥ 50% by fulfilling at least five quality requirements, a “low risk of bias” was considered.

## Results

### Selected studies

**Fig. 1:**
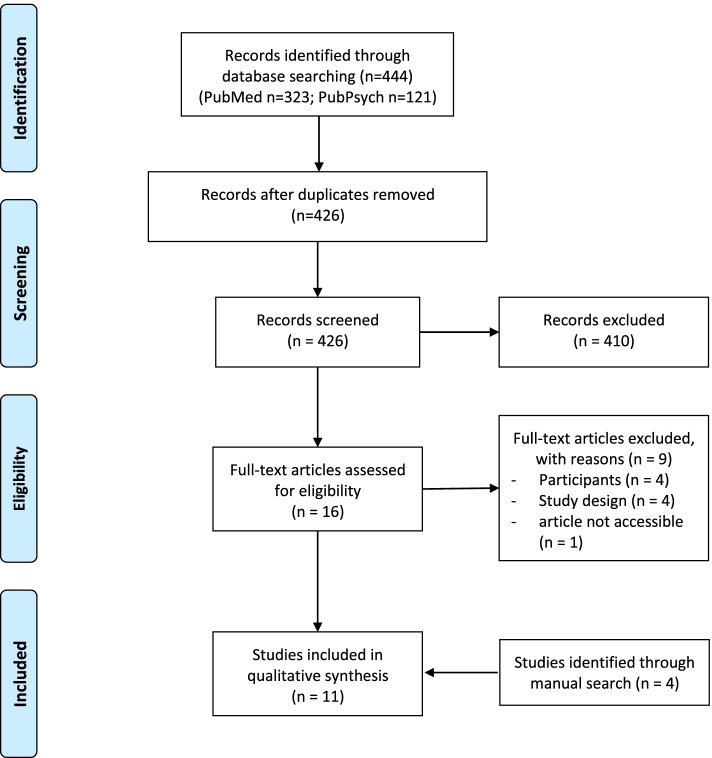
PRISMA Flow chart of the systematic literature search

During the initial search 444 publications were identified. After duplicates’ removal 426 publications were included in further screening. After screening titles and abstracts, 16 full-texts were again considered, of which seven were included in the analysis. In addition, four studies were identified by cross-referencing, what resulted in a total of eleven studies (see Fig. 1).

### Interventions and quality criteria

The eleven included studies in the review refer to eight different research projects on WHP among nurses (see Table 2). From three different projects, two publications each were included in the review [45–50]. Based on the eleven publications, seven studies were only conducted in acute medical care hospitals [45, 46, 49–53] and three only in inpatient LTC facilities [47, 48, 54]. One study was designated as a cross-setting study (inpatient LTC facilities and home-based LTC) but due to an institutional drop out results were only available for the setting of inpatient LTC facilities [55]. In terms of outcomes, most studies solely aimed to improve mental health [47–53]. Three studies reported on interventions aiming at improving only physical health [45, 46, 54] and one study targeted both mental and physical health [55]. Violence experiences were not the content of any of the included studies.

**Table 2:** WHP interventions for nurses in Germany and quality criteria

**Author (year)**	**Setting**	**Health issue**	**Quality criteria**		
			**Planning and concept quality**	**Process quality**	**Structural quality** (provider qualification)
			a) target group	a) group size	
			b) content	b) contraindications	
			c) participant materials	c) Number/ duration/ frequency of units	
			d) Theoretical framework / evidence base of the intervention	d) Location	
Becker et al. (2017) [45] & Becker et al. (2020) [46]	acute medical care hospital	Physical health:MSC^a^	a) Nurses with MSC in the shoulder, neck, or back region	a) Physiotherapy: max. 6, coaching: 2 group sessions (max. 6), 5 single sessions	- qualified physiotherapists (*n* = 14)
			b) physiotherapy exercises (guided monitored movement therapy, seven-station programme, individual adaption), work-related psychosocial coaching (SOC^b^)	b) specific physical symptoms, serious other illnesses, further medical or therapeutic interventions parallel to the study	- certified supervisor and coach, management consultant, physiotherapist and teacher (*n* = 1)
			c) not reported	c) Physiotherapy: 10/ 45 min^c^/ weekly, coaching: 7/90 min/ every 14 days	
			d) SOC	d) Physiotherapy: local physiotherapy practices, coaching: not reported	
Bernburg et al. (2019) [51]	acute medical care hospital	Mental health: stress management	a) Nurses working in psychiatric hospital departments	a) 10–12	registered and accreditated psychotherapists
			b) stress management, coping skills	b) sickness absence	
			c) not reported	c) 12/ 90–120 min/ weekly	
			d) mindfulness and acceptance training, cognitive behavioural training, solution focused group work	d) Not reported	
Görres et al. (2013) [55]	inpatient LTC^d^ facility	Mental health: well-being, physical health: general health status	a) All employees in the facilities, focus on nurses	a) not reported	not reported
			b) Health promotion day, stress, mobbing, burnout, team building, religion, communication, conflicts, death, physical activity, back pain, addictive behaviour prevention, time management, work processes, leadership behavior	b) not reported	
			c) not reported	c) not reported	
			d) not reported	d) inhouse	
Kozak et al. (2017) [54]	inpatient LTC facility	Physical health: musculoskeletal complaints	a) in-patient geriatric nurses	a) not reported	not reported
			b) knowledge transfer on body postures in nursing professions, body awareness training and physical exercises, ergonomic practical instructions, c) reorganization and redesign	b) Senior management position, trainees, pregnancy, planning any lengthy in-service training or leave of absence, back problems that might inhibit the performance of specific care tasks	
			c) not reported	c) 2 basic seminars/ one day/ not reported, 2 follow-up seminars/ half-day/ after 8 and 12 weeks	
			d) not reported	d) Inhouse	
Maatouk et al. (2016) [49]	acute medical care hospital	Mental health: stress management	a) nurses aged > 45 years	a) 9	not reported
			b) identification of the individual stressors and modification of personal strategies to cope with stress, biographical work (focused on working life), SOC training, age stereotype work, relaxation exercises	b) not reported	
			c) stress prevention CD^e^	c) 10/ 90 min/ weekly	
			d) SOC	d) not reported	
Maatouk et al. (2018) [50]	acute medical care hospital	Mental health: well-being, mental health-related quality of life	a) nursing employees aged > 45	a) Approximately 10	Two trainers (minimum qualification: a degree in medicine or psychology and training or experience in psychotherapy/ group leading with a working experience of at least two years)
			b) Introduction to the subject “ageing in care professions”, reflecting the working biography, coping with stress and the concept of mindfulness, SOC focused sessions	b) Membership in management team, leadership position, occupational disability, cognitive impairment, serious physical or psychiatric illnesses	
			c) Not reported	c) 8/ 120 min/ 7 weekly sessions, 1 booster session after 6 weeks	
			d) health belief model, trans-theoretical model of Behavior Change, social cognitive theory, transactional model of stress, SOC	d) Inhouse, during working time	
Müller et al. (2016) [52]	acute medical care hospital	Mental health: well-being	a) nurses	a) 6–8	female experienced occupational health professional (*n* = 1) and female student assistant (*n* = 1)
			b) stress and well-being in the workplace, SOC, SMART goal setting, action plan development, implementation and adaptation, reflection	b) not reported	
			c) manuals with information on work stress, SOC, goal selection, action planning, worksheets, diary to monitor the personal projects	c) 6/ 1. session: 8 h, 2. -4. & 6. session: 120 min, 5. session: 30 min/ 1. interval: 2 weeks, 2. & 3. interval: 4 weeks, 5. & 6. interval: 8 weeks	
			d) SOC	d) Inhouse, quiet room, during working time	
Zimber et al. (2010) [48] & Gregersen et al. (2010) [47]	inpatient LTC facility	Mental health: stress management	a) Nurses and managers (sessions 1–8 identical, sessions 9–12 target group specific)	a) Max. 12	not reported
			b) Dealing with difficult residents (sessions 1–4), professional self-image, dealing with stress and personal problems (sessions 5–8), communication and leadership (sessions 9–12)	b) not reported	
			c) Not reported	c) 12/ 90 min/ weekly	
			d) Concept of key skills	d) Inhouse	
Zimber et al. (2012) [53]	acute medical care hospital	Mental health: stress management	a) nurses	a) moderator training: 19, collegial counselling: 1–12	- Moderator training: not reported
			b) moderator training, collegial counselling (patient-related topics, emotional processing of work, collegial counselling, conflicts, problems with colleagues, conflict resolution, general problems)	b) not reported	-Collegial counselling: trained moderators (nurses)
			c) not reported	c) moderator training: 4/ 1 day/ not reported, collegial counselling: 0–5/ month/ 45 min/ not reported	
			d) transactional stress theory	d) inhouse	

^a^*MSC* Musculoskeletal complaints

^b^*SOC* Selection Optimization Compensation

^c^*Min* Minutes

^d^*LCT* Long-term care

^e^*CD* Compact disc

Regarding the quality criteria assessed, information on the intervention provider’s qualification was given in about half of the studies [45, 46, 50–53]. Even though all interventions targeted nurses, some studies still addressed specific subgroups, such as nurses with physical complaints [45, 46], managerial roles [47, 48], working in a specific setting [51, 54] or being at a defined age [49, 50]. Manuals included (psychosocial) stress training programs [45–52], dealing with difficult residents, communication, and leadership [47, 48], physiotherapy exercises [45, 46], collegial counselling [53], ergonomics training [54], as well as a multi-component program [55]. Nine studies reported an underlying theoretical framework of their intervention [45–53]. Regarding the process quality, the intervention groups were designed for six [45, 46, 52] to 19 [53] participants. Contraindications for the participation were reported depending on the content of the intervention in five studies [45, 46, 50, 51, 54]. The number of intervention units varied from four [53, 54] to twelve [47, 48, 51], with a duration of the units between 45 min [45, 46, 53] and eight hours [52]. The frequency of units was mostly weekly, except in one study [52]. Seven studies were conducted as inhouse training programs [47, 48, 50, 52–55].

### Appraisal according to the RE-AIM dimensions

With regard to study design, five studies were conducted as randomized controlled trials (RCT) [45, 46, 50–52], three studies had a quasi-experimental design [47, 48, 53] and three studies as a single-group longitudinal study [49, 54, 55]. Five studies were designated as pilot studies [46, 49, 51, 53, 54]. According to the Delphi List study quality regarding the fulfilment of quality requirements for intervention studies varied from 0 to 78% (Table 3).

**Table 3:** Study design and interventions’ appraisal based on the RE-AIM framework

Author (year)	Study design	Reach	Effectiveness	Adoption	Implementation	Maintenance	Delphi Score
		a) sample size (n)					
		b) age					
		c) female (%)					
Becker et al. (2017) [45]	RCT^a^/ CG^b^: physiotherapy exercises	a) 68 (IG^c^ = 34; CG = 34)	*TxG*^*f*^	not reported	not reported	*TxG*	78%
		b) (M^d^$\pm$ SD^e^) IG: 44.41 ± 9.89; CG: 43.09 ± 10.75	functional status of the locomotor system			**3 months**	
			- restriction of muscle strength: n.s			functional status of the locomotor system	
		c) IG = 85.29; CG = 88.24	- restriction of maximum degree movement: n.s			- restriction of muscle strength: not reported	
			- restriction of everyday activities: n.s			- restriction of maximum degree movement: not reported	
			pain severity/ impairment by pain:			- restriction of everyday activities: n.s	
			- due to maximum degree movement: n.s			pain severity/ impairment by pain:	
			- on everyday movement: ↓*			- due to maximum degree movement: not reported	
			- impairment			- on every day movement: ↓*	
			due to pain: n.s			- impairment due to pain: n.s	
Becker et al. (2020) [46]	RCT pilot study / CG: physiotherapy exercises	3^rd^ follow-up	not reported	not reported	not reported	*TxG*	78%
		a) 44 (IG = 24; CG = 20)				**24 months**	
		b) (M ± SD) 43.98 ± 9.59				functional status of the locomotor system	
		c) 86.36				- restriction of muscle strength: n.s	
						- restriction of maximum degree movement: ↓*	
						- restriction of everyday activities: n.s	
						pain severity/ impairment by pain:	
						- due to maximum degree movement: n.s	
						- on everyday movement: n.s	
						- impairment due to pain: n.s	
Bernburg et al. (2019) [51]	RCT pilot study / CG: non-intervention	a) 86 (IG = 44; WCG^h^ = 42)	*TxG*	not reported	not reported	*TxG*	44%
		b) (M ± SD) IG: 31.3 ± 2.5; WCG: 32.8 ± 2.1	perceived job stress: ↓**			**6 months**: perceived job stress: ↓**	
		c) IG = 82; WCG = 79				**12 months**: perceived job stress: ↓**	
Görres et al. (2013) [55]	Longitudinal intervention study / no CG	a) 119	not reported	nine inpatient LTC^i^ facilities	not reported	not reported	0%
		b) 55% > 45 years					
		c) 85					
Kozak et al. (2017) [54]	Pre-experimental pilot study / CG: no CG	a) 22	not reported	Six inpatient LTC facilities, each with two wards	not reported	*WGD*^*j*^	22%
		b) n (%) ≤ 39 = 4 (17.3) ≤ 49 = 11 (47.8) ≤ 59 = 7 (39.1)				**6 months**: time spent in sagittal inclinations exceeding 20° ↓**, exceeding 60° ↓**, static inclinations ↓**, duration of static inclination > 20° ↓**	
		c) 100					
Maatouk et al. (2016) [49]	Pilot study / CG: no CG	a) 9	not reported	not reported	reported	not reported	0%
		b) not reported					
		c) not reported					
Maatouk et al. (2018) [50]	RCT / CG: non-intervention	a) 107 (IG = 52; WCG = 55)	*TxG* (ITT^k^)	four acute medical care hospitals	not reported	not reported	56%
		b) (M ± SD) IG: 51.62 ± 4.65; WCG: 52.6 ± 5.56	- Well-being: n.s				
		c) IG = 87; WCG = 87	- Mental health-related quality of life: ↑*				
Müller et al. (2016) [52]	RCT / CG: non-intervention	a) 70 (IG = 36; CG = 34)	*TxG*	one acute medical care hospital	not reported	not reported	56%
		b) (M ± SD) IG: 44.67 ± 9.34; CG:	- ITT with adjusted *p*-value: well-being: n.s				
		42.74 ± 9.91	- PP^l^ with adjusted *p*-value: well-being: n.s				
		c) IG = 94.4; CG = 94.1					
Zimber et al. (2010) [48] & Gregersen et al. (2010) [47]	Quasi-experimental / CG: non-intervention	a) 202 (IG = 76; CG = 126)	*TxG*	eleven inpatient LTC facilities (later implementation in about 150 inpatient LTC facilities)	reported	not reported	11%
		b) not reported	- competences (personal, professional, social, organisational, overall, internal control conviction, self efficacy): n.s				
		c) not reported	- Social ressources (relationship to residents: ↓**, climate/ communication with colleagues/with supervisor: n.s.)				
			- Work load: n.s				
			- Consequences of stress: n.s				
			- Organisational ressources: n.s				
Zimber et al. (2012) [53]	Quasi-experimental pilot study / CG: non-intervention	a) 85	*TxG*	One acute medical care hospital	not reported	not reported	11%
		b) n (%) 20–29: 18 (21.2) 30–39: 24	- Influence at work: n.s				
		(28.2) 40–49: 24 (28.2) 50–59: 13 (15.3) > 60: 2 (2.4)	- Scope for decision-making: n.s				
		c) 70.6	- Development options: n.s				
			- Social support (from colleagues: n.s., from supervisor: n.s.)				
			- Feedback (from colleagues: n.s., from supervisor: n.s.)				
			- Sense of community: ↑*				
			- Competences (methodical: n.s., social: n.s., professional self-efficacy: n.s.)				
			- Stress management (emotion-oriented: n.s., problem-oriented: n.s.)				
			- Irritation (cognitive: n.s., emotional: n.s.)				
			- Emotional exhaustion: n.s				
			- Depersonalisation: n.s				
			- Personal fulfillment: n.s				

^a^*RCT* Randomised controlled trial

^b^*CG* Control group

^c^*IG* Intervention group

^d^* M* Mean value

^e^*SD* Standard deviation

^f^*TxG* Time x group interaction effect

^g^*n.s* not significant

^h^*WCG* Waitlist control group

^i^*LTC* Long-term care

^j^*WGD* Within group differences

^k^*ITT* Intention-to-treat analysis

^l^*PP* Per protocol analysis

↑ increase, ↓ decrease * = *p* < 0.05, ** = *p* < 0.01

Regarding the *Reach*-dimension, three studies did not report or incompletely reported participants’ characteristics [47–49] (see Table 3). The sample sizes at baseline varied from 9 [49] to 202 subjects [55]. The participants’ age ranged from 31.3 [51] to 52.6 [50] years. Overall, the proportion of female nurses in the studies was between 70.6% [53] and 100% [54]. The primary outcomes of the studies in regard to the impact of the intervention assessed (*Effectiveness*), were the functional status of the locomotor system and pain severity [45, 46], perceived job stress [51], mental health-related quality of life [50], well-being [50, 52], different competences [48], as well as ressources, irritation and burnout [53]. Four studies did not report on the effectiveness of the respective intervention [46, 47, 49, 55]. The vast majority of the outcome variables examined in the studies showed no statistically significant time x group interaction effects. In some cases, significant differences were found, e.g. with regard to impairment by pain on everyday movement [45], perceived job stress [45], mental health-related quality of life [44] relationship to residents [41] or sense of community [53]. Regarding *Adoption,* seven studies [47, 48, 50, 52–55] reported the amount and type of the participating institutions. The number of institutions varied between one [52, 53] and eleven, with the targeted implementation in about 150 facilities [47]. Nine studies did not report on *Implementation.* In two studies [47, 49] it was stated that the intervention was modified (e.g., shortening of intervention period). Seven studies did not report follow-up results in order to evaluate interventions’ *Maintenance* [47–50, 52, 53, 55]. In the remaining studies, long-term changes on targeted outcomes were assessed after three [45], six [51, 54], twelve [51], and 24 months [46]. The available results indicate for example a perceived reduction of job stress after a stress management training compared to the waiting control group [51]. Regarding physical health, results on *Maintenance* indicate a sustained reduction of time in stressful trunk postures [54] as well as a reduced pain severity on everyday movement [45] and a reduced restriction of maximum degree movement [46] by the respective intervention.

## Discussion

The aim of the review was to provide an overview of the evidence of workplace health promotion interventions for nurses in Germany. Despite the social and political relevance of the nursing profession there are only very few studies evaluating WHP interventions. It was astonishing that there was no intervention study on violence prevention or dealing with experiences of violence. It was also astonishing that there was no study results on health promotion for nurses in home-based LTC. Out of eleven intervention studies included, seven were conducted in acute medical care hospitals and four studies provided results on interventions in LTC facilities. The most frequent health aim of the WHP interventions was mental health.

Despite of the massive increase in the importance of WHP in preventive health care in Germany [33–35], our results indicate a clear lack of evaluated interventions for the highly relevant target group of nurses. This lack of substansive studies on WHP for nurses goes in line with former international reviews [56–58] and refers to the number, the content and also the methodological quality of the studies. The discrepancy between the health burdens of nurses and the content of the measures as well as the lack of care setting-specific studies is also striking. For example, the high prevalence of musculoskeletal complaints among nurses [11–13] is not reflected in a corresponding high number of evaluated WHP interventions on this topic. This discrepancy also applies in particular to the important issue of violence against nurses. Despite the high prevalence of verbal and physical violence and sexual harassment against nurses [18–21], we could not identify a single intervention study that addressed this issue in the context of nurses health promotion. Even though most of the WHP interventions for nurses included in this review address the certainly very important challenge of mental health [16, 17], the lack of consideration of the setting is particularly apparent in this topic. The lack of setting-specific studies points to an insufficient consideration of organisational challenges in the implementation of target group-specific health promotion offers in nursing. This is particularly noticeable for the socially important area of home-based LTC, for which we could not find any results taking the setting into account.

On the basis of the included studies and their results, we cannot derive any concrete recommendations for setting-related health promotion measures in nursing. As in other fields, e.g. coaching approaches in prevention and rehabilitation [59], the intervention contents and forms of implementation in the individual studies are extremely heterogeneous and difficult to compare. Beyond, the reporting of the interventions is also often insufficient. For example, five studies lack information on provider quality and three studies lack information on the theoretical basis of the intervention. The focus of the respective intervention-content is primarily on the areas of competence transfer through counselling and stress management. Despite the partly very different conceptual approaches, the results of our review confirm the high potential of mental health promotion interventions for nurses with regard to the promotion of employee health [60]. Our results are thus in line with the international literature, which describes that high quality studies focusing on specific settings and the exposure to patient aggression are needed [61]. Thereby, not only behavioural aspects but also organisational aspects should be taken into account [62]. Overall, WHP in care should be multimodal and address the nine fields of action for healthy nursing. These relate to the self-image of care, a safe and healthy environment, exercise, breaks and recreation, existential issues of caregiving, communication, qualification, work-life balance and self-management [63]. Nurses themselves mainly consider the topics of stress, communication, teamwork, relaxation, back health and strengthening to be in need of attention [64] which goes hand in hand with the results of a Delphi expert consultation [65]. For home-based LTC, the possibilities of digital interventions [66] might be promising but have not yet been explored.

From a methodological perspective, intervention research in prevention and WHP still faces major challenges in terms of evidence development [67]. In this respect, it is positive that five of the included studies were conducted as randomised controlled trials [45, 46, 50–52] and four as quasi-experimental trials [47, 48, 53, 54]. However, as the exclusive focus on effectiveness evaluation in terms of external evidence is considered insufficient in the evaluation of complex interventions in prevention and health promotion [68], the RE-AIM framework offers an appropriate evaluation framework. According to the RE-AIM framework, interventions should not only be appraised according to their effectiveness, but also take into account their *Reach, Adaptation, Implementation* and *Maintenance* [42, 43]. Against the background of the RE-AIM criteria, the reporting of most studies shows several limitations, which makes it even more difficult to draw conclusions about promising interventions in health promotion for nurses. With regard to our operationalisation of the RE-AIM criteria, the lack of reporting on *Implementation*, meaning the extent to which the program is delivered as intended, is particularly noticeable, and *Adoption* (the number of participating organisations and/or settings) was also reported in only seven of the eleven studies. The different follow-up periods, which varied between three months and 24 months, also make comparability and evaluation in terms of *Maintenance* difficult and were only reported in four studies. With regard to the challenge of evidence development for WHP in nursing, the focus should be on methodologically high-quality effectiveness studies under daily conditions. However, formative process evaluations addressing the RE-AIM criteria and also qualitative studies must not be neglected as they provide important information for the context-dependent planning and implementation.

### Limitations

To our knowledge, our study is the first review on workplace health promotion for nurses in Germany. The national focus of the review is due to the specific social law basis for workplace health promotion in Germany. However, this focus is associated with the limitation that no conclusions can be drawn regarding international comparisons. The strength of the study lies in the consideration of the nursing settings, the quality criteria for prevention measures of the statutory health insurers, different health burdens and also the RE-AIM criteria. Nevertheless, our review has some limitations. Thus, very few studies could be included in the review, which are hardly comparable due to different approaches and also different reporting quality of the results. A further limitation arises from the challenge of conceptually differentiating the nursing settings. Knowing that very different patient groups are cared for within home-based LTC and LTC facilities care as well as in hospitals (e.g. people with disabilities, children, sick and healthy elderly people, etc.) and there are also “mixed forms or hybrid forms” (e.g. geropsychiatry) we have decided not to make any further distinctions. We have decided to take the perspective of WHP providers according to which there are usually programmes for employees in home-based LTC, LTC facilities as well as acute medical hospitals. Due to the small number of studies, a further target group-specific differentiation of the results (e.g. according to the qualification of the nurses or according to trainees) was not possible. Beyond, our research was limited to scientific publications. Project reports that were not published as scientific publication in one of the databases used were not taken into account.

## Conclusion

The results of our review provide an overview about the current evidence on WHP interventions for nurses in Germany. It showed a lack of interventions that are oriented towards the target group-specific health burdens, especially violence experiences, and also a lack of consideration of the specific nursing setting, in particular home-based LTC. From this, we conclude that although WHP is meanwhile recognised as a promising approach to promote health in different work settings, nurses have not yet been sufficiently addressed as a relevant target group. As part of the efforts to improve the working situation of nurses, there is an urgent need for more methodologically high-quality and target group-specific interventions for nurses, taking into account workplace-specific health burdens and setting-specific implementation challenges. From a content perspective, to ensure quality as well as sustainable implementation, the measures should comply with the quality criteria for prevention measures of the statutory health insurers. Since the health burdens in nursing are not only associated with an increased risk of long-term illness and incapacity to work [69], but also with an increased likelihood of changing professions or jobs [70], employers should also actively support corresponding evaluation studies.

## Data Availability

All data generated or analysed during this study are included in this published article.

## Abbreviations

CD: Compact disc
CG: Control group
IG: Intervention group
ITT: Intention-to-treat analysis
LTC: Long-term care
M: Mean value
Min: Minutes
MSC: Musculoskeletal complaints
NS: Not significant
PP: Per protocol analysis
PRISMA: Preferred reporting items for systematic reviews and meta-analysis protocols
RCT: Randomized controlled trial
Non-RCT: Non-randomized controlled trial
RE-AIM: Reach, effectiveness, adoption, implementation, maintenance
SD: Standard deviation
SMART: Specific, measurable, attainable, relevant, time-bound
SOC: Selection, optimization, compensation
TxG: Time x group interaction effect
WCG: Waitlist control group
WGD: Within group differences
WHP: Workplace health promotion
WHPP: Workplace health preventive program
↑: Increase
↓: Decrease
*: Significant (*p* < .05)
**: Highly significant (*p* < .01)
